# Multi-Niche Microbiota of a Desert-Adapted Lizard: 16S rRNA Profiling of *Teratoscincus roborowskii* Endemic to the Turpan Depression in Northwest China

**DOI:** 10.3390/ani15223273

**Published:** 2025-11-12

**Authors:** Xing Luo, Jinlei He, Jie Luo, Hang Xiong, Yuying Xiao, Yanqin Zhao, Xianguang Guo, Dali Chen

**Affiliations:** 1 Department of pathogenic Biology, West China School of Basic Medical Sciences and Forensic Medicine, Sichuan University, Chengdu 610041, China; 2 China-Croatia Belt and Road Joint Laboratory on Biodiversity and Ecosystem Services, Chengdu Institute of Biology, Chinese Academy of Sciences, Chengdu 610213, China

**Keywords:** microbial communities, 16S rRNA, desert adaptation, *Teratoscincus roborowskii*, functional profiling

## Abstract

The microbial communities of desert-dwelling reptiles, such as lizards, are vital for their health and adaptation, yet remain poorly understood. This study provides the first detailed analysis of the microbiome of the Turpan Wonder Gecko (*Teratoscincus roborowskii*), using 16S rRNA sequencing on samples from its gut, oral cavity and environment in China’s Turpan Depression. The results showed distinct microbial profiles across these niches. Key findings include a highly diverse gut microbiota, primarily belonging to the phyla Bacteroidota and Bacillota, as well as thermophilic Thermodesulfobacteriota, which may enhance heat tolerance. The oral microbiota was rich in Pseudomonadota, likely aiding its omnivorous diet. Environmental samples were mostly Cyanobacteriota, typical of desert soils. Gut microbes appear to be specialized in carbohydrate metabolism, while oral microbes may assist in xenobiotic degradation. These results emphasize the important role that the gecko’s microbial communities play in its survival in harsh desert conditions.

## Simple Summary

We studied the microbes living in and on the Turpan wonder gecko, a lizard from China’s extreme desert environment. We found that distinct bacterial communities exist in the gecko’s gut, oral cavity and surrounding environment. The gut microbes are specialized for digestion and may help the gecko tolerate heat, while the oral microbes could aid in processing its omnivorous diet. This research shows that these unique microbial partnerships are essential for the gecko’s survival in the harsh desert.

## 1. Introduction

Microbial communities play fundamental roles in host physiology [1] and ecosystem functioning [2,3,4,5], yet our understanding of reptilian microbiota, particularly in extreme environments, remains limited [6,7]. While mammalian, avian, and fish microbiomes have been extensively characterized, lizards—representing over 7800 species worldwide [8]—have received comparatively little attention. This knowledge gap is particularly pronounced for species inhabiting extreme environments, where host-microbe interactions may be crucial for survival.

The gut microbiota is recognized as a key contributor to host nutrition [9], immune function [10], and environmental adaptation [7,11,12]. In reptiles, microbial communities are known to vary with factors such as diet [13], temperature [14], and habitat [6,15,16], suggesting they may play important roles in ecological adaptation. However, comprehensive studies integrating multiple body sites and their environments remain scarce.

*Teratoscincus roborowskii* (Turpan Wonder Gecko) presents an ideal model to investigate microbiome adaptations to extreme conditions. Endemic to the hyperarid Turpan Depression in Northwest China, this species thrives in one of the hottest and driest environments on Earth [17]. This region ranges from −95 to −76 m below sea level, the average annual precipitation is a mere 16.4 mm, and the annual evaporation rate is astounding 3000 mm. The extreme high temperature can reach 49.6 °C, and the maximum surface temperatures can reach up to 80 °C. While previous studies have examined its ecology (e.g., [17,18]), biogeography (e.g., [19]) and genetics (e.g., [20]), with a recent study on the impact of seasonal dietary influences on the gut microbiota [21], its microbial communities—potentially critical for desert adaptation—remain nearly uncharacterized.

This study addresses this gap by profiling bacterial communities in the gut, oral cavity, and environment of *T. roborowskii* using 16S rRNA sequencing. We specifically aimed to: (i) compare microbial diversity and composition across niches, (ii) identify habitat-specific microbial signatures, and (iii) predict functional differences between communities. Our findings advance understanding of microbiome-mediated adaptations in extreme environments and establish a foundation for future studies of desert-adapted species.

## 2. Materials and Methods

### 2.1. Sample Collection

**Figure 1:**
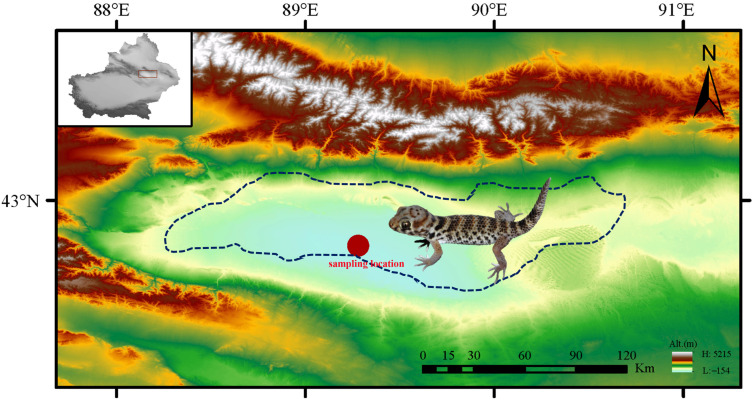
Geographic sampling location of *T. roborowskii* in this study. The dotted line indicates the species’ range in the Turpan Depression of Xinjiang. Photograph by Xianguang Guo.

On 10 June 2023, nine *T. roborowskii* geckos were captured (42.7769° N, 89.2831° E, 144 m below sea level) in the Turpan Depression, Xinjiang, China, during their peak activity hours (23:00–01:00) and fresh fecal pellets were collected during the fasting period (Figure 1). They were located using flashlight-induced eyeshine detection. After recording the gecoks’ sex, location, and developmental stage, fecal samples (FG) were collected by placing each gecko in a sterile container (1.5 L sterile mineral water bottle) and monitoring it every two hours. Fresh feces were then promptly transferred into labeled sterile EP tubes using sterile forceps and immediately frozen in liquid nitrogen to preserve sample integrity. Oral samples (OG) were collected using sterile throat swabs and stored in the same way. Four environmental samples (EG) from the geckos’ habitat were collected using the same sterile protocol. All samples were immediately frozen in liquid nitrogen. All geckos were confirmed to be in good physiological condition after the experiment and were released at the capture site, in strict adherence to animal welfare regulations. When sampling in summer to capture a specific season, it is also the hottest time of year in the Turpan Depression. *T. roborowskii* living here is therefore the best model for studying lizard adaptation to arid environments at this time.

### 2.2. DNA Extraction

Genomic DNA was extracted from the total microbial community of each sample using the E.Z.N.a™ Mag\Bind Soil DNA Kit (Omega, M5635-02, Norcross, GA, USA) according to the manufacturer’s instructions. The concentration of the extracted DNA was measured using a Qubit 3.0 fluorometer (Thermo Fisher Scientific, Waltham, MA, USA) to ensure that a sufficient amount of high-quality genomic DNA was obtained.

### 2.3. 16S rRNA Amplification and Sequencing

The V4–V5 hypervariable region of the bacterial 16S rRNA gene was targeted for amplification using universal primers 515F (GTGCCAGCMGCCGCGGTAA) and 907R (CCCCGYCAATTCMTTTRAGT) [22] in a 30 µL reaction volume containing 10–20 ng DNA template, 1 µL of each primer (10 µM), 15 µL 2× Hieff^®^ Robust PCR Master Mix (Yeasen, Shanghai, China), and nuclease-free water. Amplification was performed in an Applied Biosystems 9700 thermal cycler (Thermo Fisher Scientific, Waltham, MA, USA) using a two-step program: (1) initial denaturation at 94 °C for 3 min; 5 cycles of 94 °C for 30 s, 45 °C for 20 s, 65 °C for 30 s; then 20 cycles of 94 °C for 20 s, 55 °C for 20 s, 72 °C for 30 s; final extension at 72 °C for 5 min; followed by (2) 95 °C for 3 min; 5 cycles of 94 °C for 20 s, 55 °C for 20 s, 72 °C for 30 s; final extension at 72 °C for 5 min. PCR products were verified via 2% agarose gel electrophoresis, purified using Hieff NGS™ DNA Selection Beads (Yeasen, Shanghai, China) to remove primer dimers, quantified by Qubit^®^ dsDNA assay (Thermo Fisher, Waltham, MA, USA) and bioanalyzer (Agilent 2100, Agilent Technologies, Santa Clara, CA, USA), pooled equimolarly, and sequenced on an Illumina MiSeq platform (Illumina, San Diego, CA, USA) by Sangon BioTech (Shanghai, China) after library construction with Illumina adaptors/indices.

### 2.4. Microbial Community Analysis Pipeline

Following Illumina MiSeq paired-end sequencing, reads were assembled using PEAR (v0.9.8; [23]) based on their overlap. The resulting FASTQ files were processed into FASTA/QUAL format [24] for downstream processing. Quality-controlled (QC) sequences were clustered into Operational Taxonomic Units (OTUs) at ≥97% similarity threshold using USEARCH (v11.0.667; [25]), with chimera removal (UCHIME algorithm) and exclusion of singleton OTUs to minimize artifacts. Bacterial OTUs were classified by BLAST (v 2.10.0) against the SILVA [26] with a confidence threshold of 80% [27]. The most abundant sequence within each OTU cluster was designated as the representative sequence for annotation.

### 2.5. Statistical Analysis

Alpha diversity indices (Chao1, Simpson, Shannon) and rarefaction curves were calculated based on OTU richness using Mothur (v3.8.31; [28]). To analyze the diversity and distribution of operational taxonomic units (OTUs) in our samples, we generated rank abundance curves using the statistical software R (v4.2.2; [29]). The rank abundance curves were calculated using the rankabundance function from the vegan package (v2.6-2; [30]), which sorts the OTUs by their abundance and assigns ranks. The resulting data were then visualized using the ggplot2 package (v3.3.6; [31]), a powerful tool for creating high-quality plots in R (v3.6.0).

Within-sample (alpha) diversity comparisons between groups were performed using *t*-tests for two groups or ANOVA for multiple groups. Beta diversity (between-sample differences) was analyzed via Principal Coordinate Analysis (PCoA) and visualized using the R vegan package (v2.5-6; [30]). The PCoA was generated based on Bray–Curtis dissimilarity distance, unweighted UniFrac, and weighted UniFrac, respectively. Differential feature abundance between groups was identified using STAMP (v2.1.3; [32]) and LEfSe (v1.1.0; [33]). Microbial associations were assessed using SparCC (v1.1.0; [34]) to compute correlation coefficients and *p*-values, with results visualized as correlation heatmaps using the R corrplot package (v0.84; [35]). Co-occurrence networks were constructed using the R ggraph package (v2.2.1; [36]).

### 2.6. Function Prediction

Functional potential of bacterial and archaeal communities was predicted using PICRUSt (v1.1.4; [37]). This analysis inferred metabolic capabilities by comparing the obtained 16S rRNA gene sequencing data against a reference genome database of known functions. PICRUSt generated predictions for KEGG pathways (Kyoto Encyclopedia of Genes and Genomes) and COG pathways (Clusters of Orthologous Groups) to characterize the communities’ potential roles in metabolic processes.

## 3. Results

### 3.1. Evaluation of Microbial 16S rRNA Gene Sequencing

**Figure 2:**
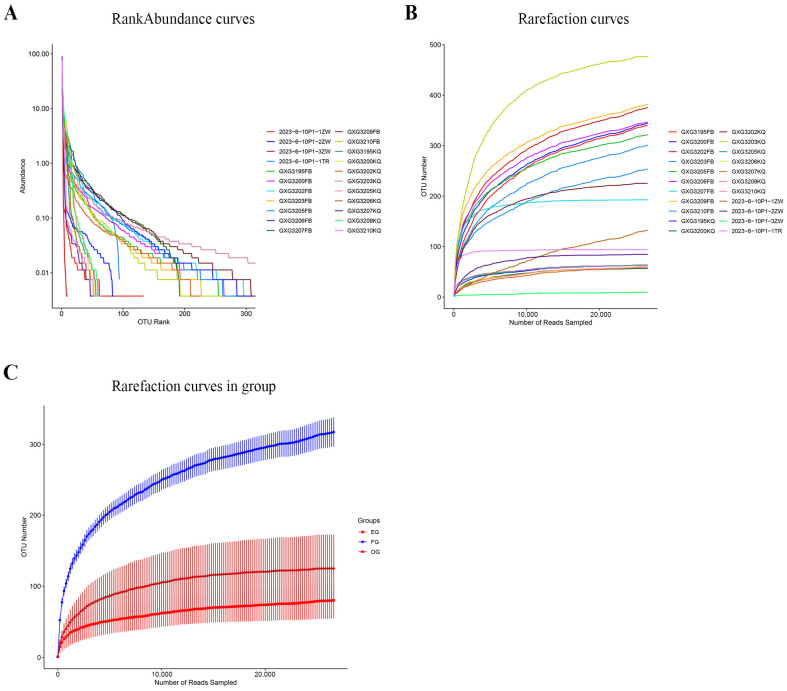
Microbial community diversity assessment. (**A**) Rank-abundance curves showing species richness and evenness across sample groups (9 fecal [FG], 9 oral [OG], and 4 environmental [EG for vegetation and TR for soil] samples). Different colors represent different samples. (**B**) Rarefaction curves demonstrating sequencing depth adequacy, with all curves approaching saturation, indicating sufficient sampling effort for community characterization. (**C**) Rarefaction curves in groups, blue indicates FG, brown indicates OG, red indicates EG. Both analyses were performed using 16S rRNA gene sequencing data.

Illumina MiSeq sequencing of 22 samples generated 2,145,644 raw reads (range: 43,994–117,111 reads per sample; average length: 350.35–414.85 bp), yielding 2,080,890 high-quality sequences (range: 31,155–165,836 reads per sample; average length: 374.04–377.71 bp) after QC. Rank Abundance Curves (Figure 2A) were analyzed to assess diversity, where the horizontal span reflects species richness (the longer the axis, the higher the richness) and the curve slope indicates evenness (the flatter the curve, the greater the evenness). The wide, gradually flattening curves observed demonstrate uniform sample composition and high species richness. Conversely, rarefaction curves (Figure 2B) are a graphical representation method used in ecology to evaluate sample completeness and explore biodiversity. By randomly selecting a certain number of sequences from each sample (i.e., resampling at a given depth that does not exceed the sequencing capacity of the samples), it is possible to predict the total number of species that may be included, as well as the relative abundance of each species at various sequencing depths. This curve helps to determine whether the sampling work is sufficient to cover most of the species present in the community. When the curve tends to flatten, this indicates that adding more samples is unlikely to reveal more species. Figure 2C shows the rarefaction curves between groups.

### 3.2. Microbial Diversity Analysis

**Figure 3:**
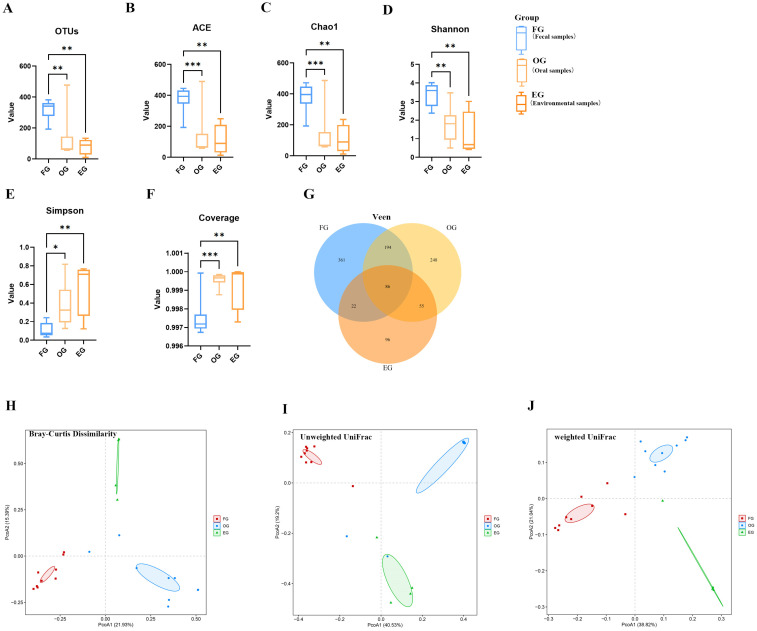
Microbial community diversity analysis. (**A**–**F**) Boxplots of α-diversity indices comparing fecal (FG), oral (OG), and environmental (EG) samples: observed OTUs (**A**), Ace (**B**), Chao1 (**C**), Shannon (**D**), Simpson (**E**), and Coverage (**F**). Significant differences are indicated (* *p* < 0.05, ** *p* < 0.01, *** *p* < 0.001), with notable divergence between FG vs. OG (*p* < 0.05) and FG vs. EG groups. (**G**) Venn diagram of shared/unique OTUs among groups. (**H**) Results of principal coordinate analysis (PCoA) based on Bray–Curtis dissimilarity, (**I**) unweighted UniFrac (community members), and (**J**) weighted UniFrac (community structure) distances.

After flattening the number of tags and performing clustering, an OTU table was generated, and 1062 OTU species were obtained. Clustering and annotation of OTUs (26 phyla, 518 species) revealed 86 shared OTUs between the fecal, oral and environmental groups, with 361, 248, and 96 unique OTUs in fecal, oral, and environmental groups, respectively (Figure 3G). Alpha diversity showed significantly higher richness (Ace, Chao1) and diversity (Shannon, Simpson) in the fecal group vs. oral/environmental groups (*p* < 0.001–0.05; Figure 3A–E), with coverage > 99.9% (Figure 3F). Beta diversity (PCoA using Bray–Curtis dissimilarity, unweighted UniFrac, and weighted UniFrac); (Figure 3H,J) confirmed the distinct clustering of fecal, oral, and environmental communities, reflecting significant compositional differences (Table 1). These results demonstrate uniquely high diversity and structural divergence of the fecal microbiome.

### 3.3. Microbial Composition Across Habitats

**Figure 4:**
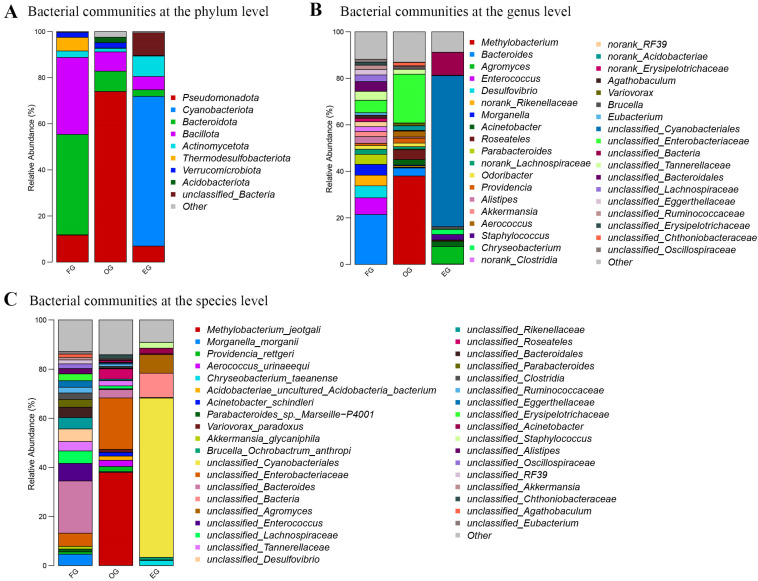
Taxonomic composition of bacterial communities in fecal (FG), oral (OG), and environmental (EG) samples. Stacked bar plots show relative abundance at (**A**) phylum, (**B**) genus, and (**C**) species levels. The *x*-axis represents individual samples; the *y*-axis shows relative abundance (%). Color blocks represent different taxa, with widths proportional to their abundance. Taxonomic classifications are displayed for each corresponding level.

Figure 4A shows the top 10 phyla with the highest content in fecal samples, oral samples and environmental samples. Fecal samples were dominated by Bacteroidota (43.52%) and Bacillota (33.41%), with notable contributions from Pseudomonadota (11.69%) and Thermodesulfobacteriota (5.91%). Oral samples exhibited extreme dominance of Pseudomonadota (73.95%), trailed distantly by Bacteroidota (8.79%) and Bacillota (8.35%), while environmental samples were overwhelmingly characterized by Cyanobacteriota (64.93%), supplemented by unclassified bacteria (9.90%), Actinomycetota (8.86%) Pseudomonadota (6.95%) and Bacillota (5.76%). Minor phyla demonstrated niche preferences—Verrucomicrobiota was ubiquitous (fecal: 2.18%, oral: 2.30%, environmental: 0.12%), whereas Acidobacteriota showed higher oral prevalence (2.35%, respectively) versus near-absence elsewhere (<0.05%).

Figure 4B shows the top 10 genera with the highest relative abundance in fecal samples, oral samples and environmental samples. Fecal samples were dominated by *Bacteroides* (21.47%) and sulfate-reducing *Desulfovibrio* (5.13%), with notable contributions from *Parabacteroides* (4.29%) and *Morganella* (4.64%), while oral samples showed remarkable dominance of *Methylobacterium* (38.01%), *Bacteroides* (3.44%) and *Enterobacteriaceae*-affiliated genera (unclassified_*Enterobacteriaceae*: 20.96%). Environmental samples were overwehelmingly composed of unclassified_*Cyanobacterales* (64.90%) and unclassified_*Bacteria* (9.90%), with *Agromyces* (7.52%) as the only major genus. Cross-habitat comparisons revealed: (1) *Bacteroides*’ prominence in feces (21.47%) versus oral samples (3.44%), (2) *Acinetobacter*’s dual presence in oral (2.41%) and environmental samples (2.24%), and (3) the exclusive environmental occurrence of *Chryseobacterium* (2.22%) and *Brucella* (1.07%). Strikingly, >70% of environmental sequences remained unclassified at genus level, underscoring the uncultured microbial diversity in this habitat.

The top 10 species with the highest content in fecal samples, oral samples and environmental samples are shown in Figure 4C. Fecal samples were dominated by unclassified_*Bacteroides* (21.28%) alongside unclassified_*Desulfovibrio* (5.13%) and pathogenic *Morganella morganii* (4.63%), demonstrating substantial taxonomic resolution gaps even for common gut microbes. Oral samples showed exceptional dominance of *Methylobacterium jeotgali* (37.98%) with notable enteric pathogens (unclassified_*Enterobacteriaceae*: 20.96%, unclassified_*Bacteroides*: 3.43%), while environmental samples remained predominantly unclassified (unclassified_*Cyanobacteriales*: 64.91%, *unclassified_Bacteria*: 9.90%, unclassified_Agromyces: 7.52%). Cross-habitat patterns included: (1) *Acinetobacter schindleri*’s presence in both oral (1.54%) and environmental (0.12%) niches, (2) the exclusive fecal occurrence of *Akkermansia_glycaniphila* (1.12%), *unclassified_Eubacterium* (1.05%) and (3) the environmental specificity of *Chryseobacterium taeanense* (2.08%). Strikingly, 87.56% of environmental sequences and 79.28% of fecal sequences lacked species-level classification, indicating substantial microbial dark matter across all habitats. The unclassified species accounted for 38.51% of the oral microbiota, which was far less than in the gut microbiota and the environmental microbiota groups.

Twelve phyla were common to all groups, with Pseudomonadota showing dramatic niche variation (gut: 11.69%, oral: 73.95%, environmental: 6.95%), while Bacteroidota dominated gut samples (43.52%) and Cyanobacteriota peaked in environmental samples (64.93%). Among 60 shared families, Enterobacteriaceae exhibited oral preference (21.08% vs. gut: 5.56%), whereas Rikenellaceae (gut: 7.87%) and Lachnospiraceae (gut: 5.99%) remained primarily gut-associated. Only one of 90 shared genera maintained > 0.1% abundance across all habitats: unclassified Enterobacteriaceae (oral: 20.96% > gut: 5.35% > environment: 0.17%), highlighting both the enteric origin of shared taxa and the strong habitat filtering of microbial communities.

### 3.4. LEfSe Analysis

**Figure 5:**
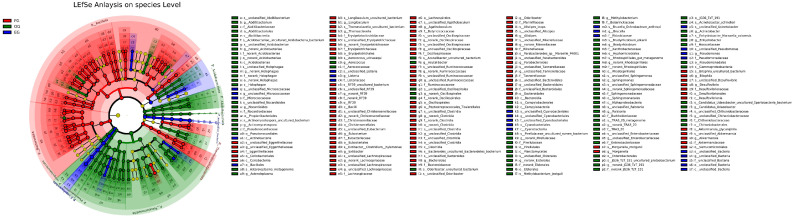
LDP evolutionary branch diagram showing biomarkers with LDA > 3 (*p* < 0.05) distinguishing fecal (FG, red), oral (OG, green), and environmental (EG, blue) groups in *T. roborowskii*. Concentric circles represent taxonomic levels from phylum (innermost) to species (outermost), with circle diameters proportional to relative abundance (p: phylum; c: class; o: order; f: family; g: genus; s: species).

Linear discriminant effect size (LEfSe) analysis (LDA score > 3) identified distinct biomarkers that validated and expanded upon the relative abundance trends: the gut microbiota was uniquely characterized by *Bacteroidales* (consistent with its 43.52% fecal dominance), while the oral microbiome showed specific enrichment of Pseudomonadota (matching its 73.95% oral prevalence), and environmental samples were distinctly marked by Cyanobacteriales (aligning with their 64.93% environmental abundance). Figure 5 shows f_norank_Acidobacteriae in the intestinal group, o_norank_Holophage in the oral group, and c_Acidobacteriae is prevalent in the environmental group. These results not only confirmed the habitat-specific patterns observed in taxonomic composition analyses but also highlighted potential functional adaptations—with Bacteroidales likely supporting gut metabolic functions, Pseudomonadota reflecting aerobic oral conditions, and Cyanobacteriales representing environmental photosynthetic niches. The robust concordance between LEfSe biomarkers and relative abundance data (Figure S1) reinforces the ecological specialization of microbial communities across these distinct habitats.

**Figure 6:**
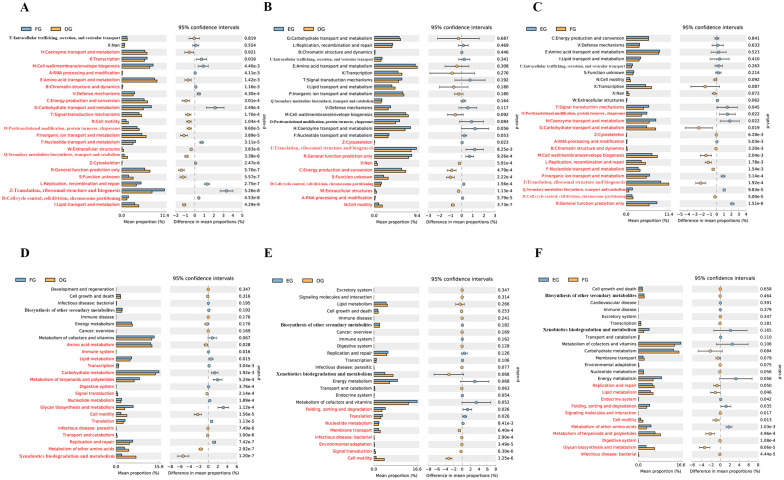
Comparative analysis of second-level metabolic pathways reveals significant differences (*p* < 0.05, red) between sample groups: (**A**–**C**) COG pathways comparing FG vs. OG (**A**), FG vs. EG (**B**), and OG vs. EG (**C**); (**D**–**F**) KEGG pathways comparing FG vs. OG (**D**), FG vs. EG (**E**), and OG vs. EG (**F**). Vertical axes indicate metabolic pathways; horizontal axes show relative proportions of each pathway.

Functional profiling using PICRUSt2 revealed distinct metabolic patterns across gut (FG), oral (OG), and environmental (EG) microbiota (Figure 6A–F and Figure S2). COG analysis showed that all three communities were dominated by metabolic functions (42.1–44.5%), but exhibited key differences: the gut microbiota had higher information processing capacity (23.9% vs. 18.4% in others) and lower proportion of poorly characterized (7.98%) functions (Table S1). Differential COG analysis (Figure 6A–C) identified oral-specific enhancements in amino acid/coenzyme transport and energy conversion, gut-specific advantages in carbohydrate/nucleotide metabolism and cell division, and environmental specialization in defense mechanisms and protein turnover.

KEGG pathway analysis further highlighted habitat-specific adaptations (Figure 6D–F). Oral microbiota showed significant enrichment in xenobiotic biodegradation and cell motility compared to gut flora. Gut communities demonstrated stronger membrane transport, glycan biosynthesis, and carbohydrate/lipid metabolism than environmental samples. Environmental microbiota exhibited enhanced nucleotide metabolism, energy production, and cofactor/vitamin synthesis compared to oral communities. These functional differences align with and help explain the observed taxonomic variations, revealing how each habitat selects for distinct metabolic capabilities.

Notably, environmental samples exhibited particularly high genetic information processing capacity (14.1% vs. 11.6% in the oral cavity) (Table S2), likely reflecting their need to adapt to variable conditions. The gut’s emphasis on carbohydrate metabolism and glycan biosynthesis underscores its role in host nutrition, while the capacity of oral flora for xenobiotic degradation suggests adaptation to dietary and environmental chemicals. The gut’s emphasis on carbohydrate metabolism and glycan biosynthesis underscores its role in host nutrition, while oral flora’s xenobiotic degradation capacity suggests adaptation to dietary and environmental chemicals. These results collectively demonstrate how microbial communities functionally specialize to thrive in their specific habitats. Together, these results demonstrate how microbial communities functionally specialize to thrive in their specific habitats.

## 4. Discussion

Environmental changes can drive rapid evolution in species that often rely on phenotypic plasticity for survival [38]. A growing body of evidence suggests that the symbiotic microbiome plays a crucial role in facilitating this plasticity and rapid environmental adaptation [39,40]. Reptiles are one of the most species-rich groups of vertebrates and are ubiquitous across global ecosystems. However, the gut and oral microbiota of reptiles such as lizards, snakes, and turtles remain understudied. The strictly nocturnal, egg-laying lizard *T. roborowskii*, which is endemic to the hyperarid Turpan Depression below sea level, provides an ideal model for investigating how the microbiome contributes to survival in extreme environments.

The gut microbiota has a profound influence on host physiology, impacting immunity [10], behavior [41], metabolism, and nutrition [9]. This community is highly sensitive to environmental factors like temperature [14], diet [6,13,42], and season [21]. As environmental changes often alter the host’s diet, they can subsequently reshape the gut microbiota, thereby modulating the host’s potential for environmental adaptation [43].

Analysis of 16S rRNA sequences revealed that the gut microbiome of *T. roborowskii* was dominated by Bacteroidota (43.52%), Bacillota (formerly the Firmicutes) (33.41%), Pseudomonadota (formerly the Proteobacteria) (11.69%), Thermodesulfobacteriota (5.91%), Actinobacteria (2.79%), and Verrucomicrobiota (2.18%). This core phylum-level composition—Bacteroidota, Bacillota, Pseudomonadota, Actinobacteria, and Verrucomicrobiota—is consistent with findings from lizards in both cold and warm regions [14] and other species like *Japalura*, iguanas, and *Diploderma* [7,44,45], indicating functional conservation. *Bacteroidota* and *Bacillota* are essential for carbohydrate fermentation and polysaccharide degradation in non-mammalian vertebrates [46,47]. *Pseudomonadota*, prevalent in carnivorous lizards, contribute to polysaccharide and protein metabolism and aromatic compound degradation [9,15,16,48,49,50,51]. These functions are critical for digestion, as hosts lack enzymes to break down many complex polysaccharides. Microbiota like Bacteroidetes supplement host capabilities by producing enzymes (e.g., xylanases, CAZymes [52,53]) that degrade indigestible dietary polymers and host-derived carbohydrates [54].

While the top three phyla remained consistent across seasons, their relative abundances and the composition of the top 10 phyla varied significantly, reflecting known influences of geography, season, and diet [21,55]. Notably, the Thermodesulfobacteriota phylum (5.96%), specifically the thermophilic, sulfate-reducing Desulfobacterota class, was prominent. This phylum, also found in heat-adapted lizards like *Diploderma* and *Japalura*, is linked to arsenic methylation and thrives at elevated temperatures [56,57]. Its presence in *T. roborowskii* suggests a potential role in host adaptation to desert thermal extremes.

As the second-largest symbiotic microbial community, the oral microbiome can influence distal gut microbiota through the oral–gut axis, thereby affecting host health [58]. The oral microbiota changes with host health status [59], developmental stage [60], diet, and population [61]. In *T. roborowskii*, the oral microbiome was distinct and dominated by *Pseudomonadota*. Although the oral microbiota of other lizards, such as *Japalura*, is dominated by Pseudomonadota, Bacteroidota, Firmicutes, Actinobacteriota, and Acidobacteriota [45], the relative abundances of Thermodesulfobacteriota and Verrucomicrobiota differ in *T. roborowskii*. Interestingly, Verrucomicrobiota has been found in the oral cavity of *Caretta caretta*, despite being unreported in the oral cavity of some lizards [45] and snakes [62,63]. This phylum exhibits strict seasonality and contributes to the degradation of sulfated and fucosylated polysaccharides [64]. Although Verrucomicrobiota members are typically mesophilic, they inhabit extreme environments like Antarctic waters, soda lakes, and hydrothermal vents [65]. They possess potent capabilities for degrading complex polysaccharides and organic matter [47,66,67,68] and may influence host physiology by producing short-chain fatty acids (SCFAs) [69,70,71]. The presence of Verrucomicrobiota in the omnivorous *T. roborowskii* but its absence in the insectivorous *Japalura* suggests a dietary link. The oral community’s structure was more strongly influenced by environmental factors than the gut community, and it differed from both gut and environmental samples in terms of diversity and abundance.

Drylands cover 40% of the Earth’s land surface, with arid and hyperarid regions accounting for 11.5% and 6.4%, respectively [72]. Organisms in these ecosystems face severe water stress. Cyanobacteriota, a photosynthetic autotroph common in dryland soils, were present, alongside other microbes typically found in arid environments, such as Firmicutes, Bacteroidota, Acidobacteriota, and Proteobacteria [73]. These phyla endure aridity through various strategies: Cyanobacteriota and Proteobacteria utilize photoautotrophy, while Firmicutes form dormant bodies. The significant presence of unclassified bacteria (9.90%) underscores the vast unknown microbial diversity in hyperarid environments, and studying this could reveal novel mechanisms of microbial resistance.

The dominant phyla present in gut, oral, and environmental samples (>1% abundance) were Bacteroidota, Bacillota, Pseudomonadota, Actinomycetota, and Verrucomicrobiota. These are primarily involved in polysaccharide and protein metabolism and organic decay. Although Thermodesulfobacteria were less prevalent in oral and environmental samples, the oral microbiota can influence the distribution of gut microbiota and consequently host physiology.

Functional predictions (KEGG pathway analysis) supported these niche-specific roles. Oral flora was enriched in pathways for initial food processing, including xenobiotic biodegradation, amino acid metabolism, and cell motility. In contrast, the gut flora, dominated by the phyla Bacteroidota and Bacillota, showed enrichment in pathways for nutrient assimilation (carbohydrate, lipid, and nucleotide metabolism) and the processing of complex compounds (glycan biosynthesis, metabolism of terpenoids and polyketides). Gut microbiota also showed enrichment in core cellular functions like translation and replication, aligning with its central metabolic role.

Overall, our results identify a core microbiome of Bacteroidota, Bacillota, Pseudomonadota, and Actinomycetota with distinct niche-specific variations. The high functional diversity of the gut microbiome aligns with its role in nutrient processing, while Pseudomonadota dominance in the oral cavity may reflect an aerobic niche. The presence of Thermodesulfobacteriota in the gut suggests thermophilic adaptations, and Verrucomicrobiota in the oral cavity may aid polysaccharide digestion in an omnivore. These findings underscore the microbiome’s critical role in desert adaptation and establish *T. roborowskii* as a key model for extremophile-host symbiosis.

## 5. Limitations

This functional analysis of this study, which is based on 16S rRNA amplicon sequencing and PICRUSt2 prediction, is inherently limited by database bias and phylogenetic inference. Techniques like metagenomic shotgun sequencing and metatranscriptomics would provide more robust functional validation. Furthermore, sampling at a single time point constrains our understanding of temporal dynamics. Future longitudinal studies tracking seasonal microbial changes will be essential. Initially using the RDP database also posed limitations; while switching to SILVA improved classification. However, a significant portion of the community remains unclassified, highlighting the novel microbial diversity in this extreme habitat awaiting discovery.

## 6. Conclusions

The Turpan wonder gecko (*T. roborowskii*), a nocturnal lizard from China’s Turpan Depression, offers valuable insights into microbiome-host symbiosis in extreme deserts. This study focuses on the gecko’s gut microbiome, which is dominated by Bacteroidota, Bacillota, and Pseudomonadota. These aid nutrient processing and heat tolerance. The oral microbiota, rich in Pseudomonadota, supports detoxification and initial food processing. Environmental samples reveal the presence of Cyanobacteriota and Firmicutes, which are typical of arid ecosystems. Functional analyses show gut microbes excel in metabolism, while oral microbes specialize in xenobiotic degradation. These findings underscore the role of the microbiome in desert adaptation, establishing *T. roborowskii* as a model for extremophile-host relationships.

## Figures and Tables

**Table 1:** Permutational multivariate analysis of variance (PERMANOVA) of microbiota samples (fecal, oral, and environmental microbiota) based on Bray–Curtis dissimilarity, unweighted UniFrac, and weighted UniFrac distances of the same host lizard species (*T. roborowskii*).

	Bray–Curtis			Unweighted UniFrac			Weighted UniFrac		
Group	F	$R^{2}$	*p*	F	$R^{2}$	*p*	F	$R^{2}$	*p*
FG vs. OG	6.916091	0.301801	0.001	15.87053	0.497969	0.001	14.08823	0.468231	0.001
FG vs. EG	5.171305	0.319783	0.002	7.654786	0.410339	0.003	14.88895	0.575108	0.002
OG vs. EG	4.384139	0.284978	0.001	4.578687	0.293907	0.005	9.050716	0.451391	0.003

## Data Availability

The raw sequence data reported in this paper have been deposited in the Genome Sequence Archive (Genomics, Proteomics & Bioinformatics 2021) in National Genomics Data Center (Nucleic Acids Res 2025), China National Center for Bioinformation/Beijing Institute of Genomics, Chinese Academy of Sciences (GSA: CRA026392) that are publicly accessible at https://ngdc.cncb.ac.cn/gsa, accessed on 9 November 2025.

## Supplementary Materials

The following supporting information can be downloaded at: https://www.mdpi.com/article/10.3390/ani15223273/s1, Figure S1. LEfSe-based microbial community analysis of *T. roborowskii*. The figure shows bacterial taxa with significantly differential abundance (LDA score > 5, *p* < 0.05) in fecal (FG, red), oral (OG, green), and environmental (EG, blue) groups. Taxonomic levels: phylum (p), class (c), order (o), family (f), genus (g), species (s). Functional Specialization Across Gut, Oral, and Environmental Microbiota; Figure S2. Differential metabolic pathway analysis of *T. roborowskii* comparing fecal (FG), oral (OG), and environmental (EG) microbiomes using COG and KEGG annotations: (A–C) COG and (D–F) KEGG bar plots show significantly divergent first-level pathways (*p* < 0.05, red bars) between FG vs. OG (A,D), OG vs. EG (B,E), and FG vs. EG (C,F), with *y*-axes indicating pathways and *x*-axes showing relative proportions.; Table S1. Results of COG analyses predicted using PICRUSt; Table S2. Results of KEGG analyses predicted using PICRUSt.

## References

[1] Shang Y., Zhong H., Liu G., Wang X., Wu X., Wei Q., Shi L., Zhang H. (2023). Characteristics of flora in different segments of the digestive tract of *Lycodon rufozonatus*. Animals.

[2] Acharyya S., Majumder S., Nandi S., Ghosh A., Saha S., Bhattacharya M. (2025). Uncovering mercury accumulation and the potential for bacterial bioremediation in response to contamination in the Singalila National Park. Sci. Rep..

[3] Duffy J.E., Godwin C.M., Cardinale B.J. (2017). Biodiversity effects in the wild are common and as strong as key drivers of productivity. Nature.

[4] Huang Z.K., Dao C.J., Ma P.X., Li B., Yan K. (2025). Characteristics of nitrogen cycle-related bacterial community and its response to soil in the main lead-zinc mine reclamation area of Lanping. Huan Jing Ke Xue.

[5] Naeem S., Duffy J.E., Zavaleta E. (2012). The functions of biological diversity in an age of extinction. Science.

[6] Tang S., Li Y., Huang C., Yan S., Li Y., Chen Z., Wu Z. (2022). Comparison of gut flora diversity between captive and wild Tokay gecko (*Gekko gecko*). Front. Microbiol..

[7] Zhu W., Qi Y., Wang X., Shi X., Chang L., Liu J., Zhu L., Jiang J. (2022). Multi-omics approaches revealed the associations of host metabolism and gut microbiome with phylogeny and environmental adaptation in mountain dragons. Front. Microbiol..

[8] Uetz P., Freed P., Aguilar R., Reyes F., Kudera J., Hošek J. The Reptile Database. https://reptile-database.reptarium.cz/search.

[9] Fung T.C., Olson C.A., Hsiao E.Y. (2017). Interactions between the microbiota, immune and nervous systems in health and disease. Nat. Neurosci..

[10] Belkaid Y., Hand T.W. (2014). Role of the microbiota in immunity and inflammation. Cell.

[11] Kim P.S., Shin N.R., Lee J.B., Kim M.S., Whon T.W., Hyun D.W., Yun J.H., Jung M.J., Kim J.Y., Bae J.W. (2021). Host habitat is the major determinant of the gut microbiome of fish. Microbiome.

[12] Xiao F., Zhu W., Yu Y., He Z., Wu B., Wang C., Shu L., Li X., Yin H., Wang J. (2021). Host development overwhelms environmental dispersal in governing the ecological succession of zebrafish gut flora. NPJ Biofilms Microbiomes.

[13] Jiang H.Y., Ma J.E., Li J., Zhang X.J., Li L.M., He N., Liu H.Y., Luo S.Y., Wu Z.J., Han R.C. (2017). Diets alter the gut microbiome of crocodile lizards. Front. Microbiol..

[14] Zhu X.M., Chen J.Q., Du Y., Lin C.X., Qu Y.F., Lin L.H., Ji X. (2024). Microbial communities are thermally more sensitive in warm-climate lizards compared with their cold-climate counterparts. Front. Microbiol..

[15] Zhang W., Li N., Tang X., Liu N., Zhao W. (2018). Changes in intestinal flora across an altitudinal gradient in the lizard *Phrynocephalus vlangalii*. Ecol. Evol..

[16] Zhang L., Yang F., Li N., Dayananda B. (2021). Environment-dependent variation in gut flora of an oviparous lizard (*Calotes versicolor*). Animals.

[17] Zhao E.M., Zhao K.T., Zhou K.Y. (1999). Fauna Sinica, Reptilia, Vol. 2, Squamata, Lacertilia.

[18] Li W.R., Song Y.C., Shi L. (2013). Home range of *Teratoscincus roborowskii* (Gekkonidae): Influence of sex, season, and body size. Acta Ecol. Sin..

[19] Macey J.R., Wang Y., Ananjeva N.B., Larson A., Papenfuss T.J. (1999). Vicariant patterns of fragmentation among gekkonid lizards of the genus *Teratoscincus* produced by the Indian collision: A molecular phylogenetic perspective and an area cladogram for Central Asia. Mol. Phylogenet. Evol..

[20] Zheng D., Ma R., Guo X., Li J. (2025). Comparative mitogenomics of wonder geckos (Sphaerodactylidae: *Teratoscincus* Strauch, 1863): Uncovering evolutionary insights into protein-coding genes. Genes.

[21] Gao W.Z., Yang Y., Shi L. (2023). Seasonal dietary shifts alter the gut flora of a frugivorous lizard *T. roborowskii* (Squamata, Sphaerodactylidae). Ecol. Evol..

[22] Jiang G.H., Li H.Y., Xie L.J., Fan J.Y., Li S.Y., Yu W.Q., Xu Y.T., He M.L., Jiang Y., Bai X. (2025). Intestinal flora was associated with occurrence risk of chronic non-communicable diseases. World J. Gastroenterol..

[23] Zhang J., Kobert K., Flouri T., Stamatakis A. (2014). PEAR: A fast and accurate Illumina Paired-End reAd mergeR. Bioinformatics.

[24] Schmieder R., Edwards R. (2011). Quality control and preprocessing of metagenomic datasets. Bioinformatics.

[25] Edgar R.C. (2013). UPARSE: Highly accurate OTU sequences from microbial amplicon reads. Nat. Methods.

[26] Quast C., Pruesse E., Yilmaz P., Gerken J., Schweer T., Yarza P., Peplies J., Glöckner F.O. (2013). The SILVA ribosomal RNA gene database project: Improved data processing and web-based tools. Nucleic Acids Res..

[27] Kõljalg U., Nilsson R.H., Abarenkov K., Tedersoo L., Taylor A.F.S., Bahram M., Larsson K.-H. (2013). Towards a unified paradigm for sequence-based identification of Fungi. Mol. Ecol..

[28] Schloss P.D., Westcott S.L., Ryabin T., Hall J.R., Hartmann M., Hollister E.B., Lesniewski R.A., Oakley B.B., Parks D.H., Robinson C.J. (2009). Introducing mothur: Open-source, platform-independent, community-supported software for describing and comparing microbial communities. Appl. Environ. Microbiol..

[29] R Core Team (2022). R: A Language and Environment for Statistical Computing.

[30] Oksanen J., Simpson G.L., Blanchet G.F., Kindt R., Legendre P., Minchin P.R., O’Hara R.B., Solymos P., Stevens M.H.H., Szoecs E. (2023). Vegan: Community Ecology Package. R Package.

[31] Wickham H. (2016). ggplot2: Elegant Graphics for Data Analysis.

[32] Parks D.H., Tyson G.W., Hugenholtz P., Beiko R.G. (2014). STAMP: Statistical analysis of taxonomic and functional profiles. Bioinformatics.

[33] Segata N., Izard J., Waldron L., Gevers D., Miropolsky L., Garrett W.S., Huttenhower C. (2011). Metagenomic biomarker discovery and explanation. Genome Biol..

[34] Friedman J., Alm E.J. (2012). Inferring correlation networks from genomic survey data. PLoS Comput. Biol..

[35] Calleros L., Barcellos M., Grecco S., Garzón J.P., Lozano J., Urioste V., Gastal G. (2024). Longitudinal study of the bovine cervico-vaginal bacterial microbiota throughout pregnancy using 16S ribosomal RNA gene sequences. Infect. Genet. Evol..

[36] Pedersen T.L. (2024). ggraph: An Implementation of Grammar of Graphics for Graphs and Networks. R Package.

[37] Langille M.G., Zaneveld J., Caporaso J.G., McDonald D., Knights D., Reyes J.A., Clemente J.C., Burkepile D.E., Vega Thurber R.L., Knight R. (2013). Predictive functional profiling of microbial communities using 16S rRNA marker gene sequences. Nat. Biotechnol..

[38] Littleford-Colquhoun B.L., Clemente C., Whiting M.J., Ortiz-Barrientos D., Frère C.H. (2017). Archipelagos of the Anthropocene: Rapid and extensive differentiation of native terrestrial vertebrates in a single metropolis. Mol. Ecol..

[39] Alberdi A., Aizpurua O., Bohmann K., Zepeda-Mendoza M.L., Gilbert M.T.P. (2016). Do Vertebrate Gut Metagenomes Confer Rapid Ecological Adaptation?. Trends Ecol. Evol..

[40] Littleford-Colquhoun B.L., Weyrich L.S., Kent N., Frere C.H. (2019). City life alters the gut microbiome and stable isotope profiling of the eastern water dragon (*Intellagama lesueurii*). Mol. Ecol..

[41] Needham B.D., Funabashi M., Adame M.D., Wang Z., Boktor J.C., Haney J., Wu W.L., Rabut C., Ladinsky M.S., Hwang S.J. (2022). A gut-derived metabolite alters brain activity and anxiety behaviour in mice. Nature.

[42] Zhou J., Zhao Y.T., Dai Y.Y., Jiang Y.J., Lin L.H., Li H., Li P., Qu Y.F., Ji X. (2020). Captivity affects diversity, abundance, and functional pathways of gut flora in the northern grass lizard *Takydromus septentrionalis*. Microbiologyopen.

[43] De Filippo C., Cavalieri D., Di Paola M., Ramazzotti M., Poullet J.B., Massart S., Collini S., Pieraccini G., Lionetti P. (2010). Impact of diet in shaping gut microbiota revealed by a comparative study in children from Europe and rural Africa. Proc. Natl. Acad. Sci. USA.

[44] Hong P.Y., Wheeler E., Cann I.K., Mackie R.I. (2011). Phylogenetic analysis of the fecal microbial community in herbivorous land and marine iguanas of the Galápagos Islands using 16S rRNA-based pyrosequencing. ISME J..

[45] Tian Z., Pu H., Cai D., Luo G., Zhao L., Li K., Zou J., Zhao X., Yu M., Wu Y. (2022). Characterization of the bacterial microbiota in different gut and oral compartments of splendid japalure (*Japalura* sensu lato). BMC Vet. Res..

[46] Colston T.J., Jackson C.R. (2016). Microbiome evolution along divergent branches of the vertebrate tree of life: What is known and unknown. Mol. Ecol..

[47] Sichert A., Corzett C.H., Schechter M.S., Unfried F., Markert S., Becher D., Fernandez-Guerra A., Liebeke M., Schweder T., Polz M.F. (2020). Verrucomicrobia use hundreds of enzymes to digest the algal polysaccharide fucoidan. Nat. Microbiol..

[48] Kohl K.D., Amaya J., Passement C.A., Dearing M.D., McCue M.D. (2014). Unique and shared responses of the gut flora to prolonged fasting: A comparative study across five classes of vertebrate hosts. FEMS Microbiol. Ecol..

[49] Abdul Rahman N., Parks D.H., Vanwonterghem I., Morrison M., Tyson G.W., Hugenholtz P. (2016). A phylogenomic analysis of the bacterial phylum Fibrobacteres. Front. Microbiol..

[50] Reid N.M., Addison S.L., Macdonald L.J., Lloyd-Jones G. (2011). Biodiversity of active and inactive bacteria in the gut flora of wood-feeding huhu beetle larvae (*Prionoplus reticularis*). Appl. Environ. Microbiol..

[51] Vacca M., Celano G., Calabrese F.M., Portincasa P., Gobbetti M., De Angelis M. (2020). The controversial role of human gut Lachnospiraceae. Microorganisms.

[52] Dutschei T., Beidler I., Bartosik D., Seeßelberg J.M., Teune M., Bäumgen M., Ferreira S.Q., Heldmann J., Nagel F., Krull J. (2023). Marine Bacteroidetes enzymatically digest xylans from terrestrial plants. Environ. Microbiol..

[53] Chen J., Robb C.S., Unfried F., Kappelmann L., Markert S., Song T., Harder J., Avcı B., Becher D., Xie P. (2018). Alpha- and beta-mannan utilization by marine Bacteroidetes. Environ. Microbiol..

[54] Thomas F., Hehemann J.H., Rebuffet E., Czjzek M., Michel G. (2011). Environmental and gut bacteroidetes: The food connection. Front. Microbiol..

[55] Kohl K.D., Brun A., Magallanes M., Brinkerhoff J., Laspiur A., Acosta J.C., Caviedes-Vidal E., Bordenstein S.R. (2017). Gut microbial ecology of lizards: Insights into diversity in the wild, effects of captivity, variation across gut regions and transmission. Mol. Ecol..

[56] Wang L., Pei H., Xing T., Chen D., Chen Y., Hao Z., Tian Y., Ding J. (2025). Gut bacteria and host metabolism: The keys to sea cucumber (*Apostichopus japonicus*) quality traits. Food Chem..

[57] Yu T., Luo Y., Tan X., Zhao D., Bi X., Li C., Zheng Y., Xiang H., Hu S. (2024). Global marine cold seep metagenomes reveal diversity of taxonomy, metabolic function, and natural products. Genom. Proteom. Bionf..

[58] Lam G.A., Albarrak H., McColl C.J., Pizarro A., Sanaka H., Gomez-Nguyen A., Cominelli F., Paes Batista da Silva A. (2023). The oral-gut axis: Periodontal diseases and gastrointestinal disorders. Inflamm. Bowel. Dis..

[59] Matějková T., Hájková P., Stopková R., Stanko M., Martin J.F., Kreisinger J., Stopka P. (2020). Oral and vaginal microbiota in selected field mice of the genus *Apodemus*: A wild population study. Sci. Rep..

[60] Amin N., Schwarzkopf S., Kinoshita A., Tröscher-Mußotter J., Dänicke S., Camarinha-Silva A., Huber K., Frahm J., Seifert J. (2021). Evolution of rumen and oral microbiota in calves is influenced by age and time of weaning. Anim. Microbiome.

[61] Older C.E., Diesel A.B., Lawhon S.D., Queiroz C.R.R., Henker L.C., Rodrigues Hoffmann A. (2019). The feline cutaneous and oral microbiota are influenced by breed and environment. PLoS ONE.

[62] Larréché S., Bousquet A., da Silva L., Planelles A., Ksas R., Mérens A., Chippaux J.P. (2023). Antibiotic susceptibility of cultivable microbiota from the oral cavity of captive Bothrops atrox and Bothrops lanceolatus: Implications for the treatment of snakebite-associated infections in the French departments of America. Infect. Dis. Now.

[63] Hu X., Yang L., Zhang Y., Yang M., Li J., Fan Y., Guo P., Tian Z. (2024). Fecal and oral microbiome analysis of snakes from China reveals a novel natural emerging disease reservoir. Front. Microbiol..

[64] Orellana L.H., Francis T.B., Ferraro M., Hehemann J.H., Fuchs B.M., Amann R.I. (2022). Verrucomicrobiota are specialist consumers of sulfated methyl pentoses during diatom blooms. ISME J..

[65] Podosokorskaya O.A., Elcheninov A.G., Novikov A.A., Merkel A.Y., Kublanov I.V. (2023). *Fontisphaera persica* gen. nov., sp. nov., a thermophilic hydrolytic bacterium from a hot spring of Baikallake region, and proposal of Fontisphaeraceae fam. nov., and Limisphaeraceae fam. nov. within the *Limisphaerales* ord. nov. (*Verrucomicrobiota*). Syst. Appl. Microbiol..

[66] Gong H., Shi Y., Zhou X., Wu C., Cao P., Xu C., Hou D., Wang Y., Zhou L. (2014). Flora in the throat and risk factors for Laryngeal Carcinoma. Appl. Environ. Microbiol..

[67] Naumoff D.G., Dedysh S.N. (2018). Bacteria from poorly studied phyla as a potential source of new enzymes: β-galactosidases from planctomycetes and verrucomicrobia. Microbiology.

[68] Tan X.-Y., Liu X.-J., Lu D.-C., Ye Y.-Q., Liu X.-Y., Yu F., Yang H., Li F., Du Z.-J., Ye M.-Q. (2025). Insights into the physiological and metabolic features of *Thalassobacterium*, a novel genus of *Verrucomicrobiota* with the potential to drive the carbon cycle. MBio.

[69] Morrison D.J., Preston T. (2016). Formation of short chain fatty acids by the gut microbiota and their impact on human metabolism. Gut Microbes.

[70] Olson C.A., Vuong H.E., Yano J.M., Liang Q.Y., Nusbaum D.J., Hsiao E.Y. (2018). The Gut Microbiota mediates the anti-seizure effects of the ketogenic diet. Cell.

[71] Ouwerkerk J.P., van der Ark K.C.H., Davids M., Claassens N.J., Finestra T.R., de Vos W.M., Belzer C. (2016). Adaptation of *Akkermansia muciniphila* to the oxic-anoxic interface of the mucus layer. Appl. Environ. Microbiol..

[72] Leung P.M., Bay S.K., Meier D.V., Chiri E., Cowan D.A., Gillor O., Woebken D., Greening C. (2020). Energetic basis of microbial growth and persistence in desert ecosystems. MSystems.

[73] Demergasso C., Neilson J.W., Tebes-Cayo C., Véliz R., Ayma D., Laubitz D., Barberán A., Chong-Díaz G., Maier R.M. (2023). Hyperarid soil microbial community response to simulated rainfall. Front. Microbiol..

