# Supplementary material for: Multi-Niche Microbiota of a Desert-Adapted Lizard: 16S rRNA Profiling of Teratoscincus roborowskii Endemic to the Turpan Depression in Northwest China
Source: Animals (Basel). 2025 Nov 12;15(22):3273. doi: 10.3390/ani15223273 (PMC12649267; doi:10.3390/ani15223273)
Supplement: Supplementary file 1 [file animals-15-03273-s001.zip › animals-3850684-supplementary.pdf]

Supplementary Figures and Tables

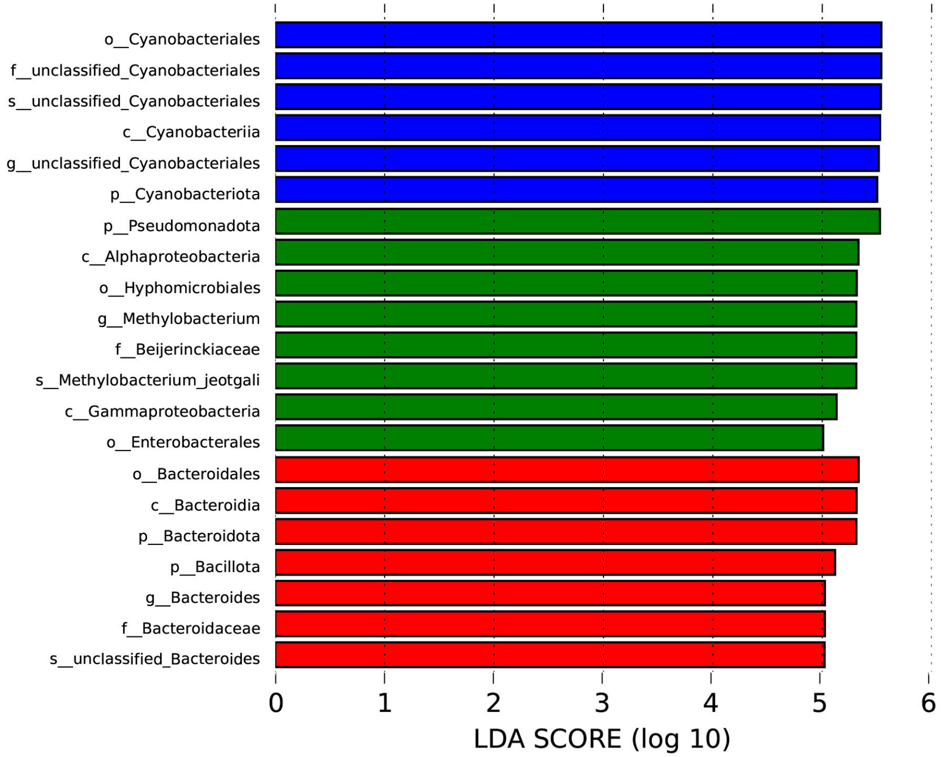

Figure S1. LEfSe-based microbial community analysis of *T. roborowskii*. The figure shows bacterial taxa with significantly differential abundance (LDA score >5, P<0.05) in fecal (FG, red), oral (OG, green), and environmental (EG, blue) groups. Taxonomic levels: phylum (p), class (c), order (o), family (f), genus (g), species (s). Functional Specialization Across Gut, Oral, and Environmental Microbiota.

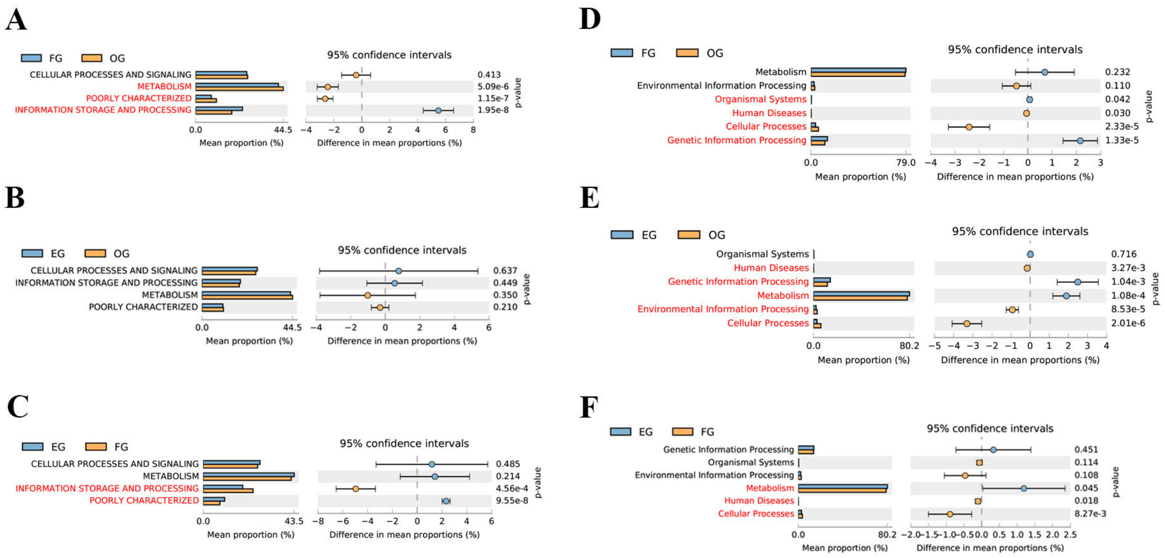

Figure S2. Differential metabolic pathway analysis of *T. roborowskii* comparing fecal (FG), oral (OG), and environmental (EG) microbiomes using COG and KEGG annotations: (A–C) COG and (D–F) KEGG bar plots show significantly divergent first-level pathways ($P < 0.05$, red bars) between FG vs. OG (A, D), OG vs. EG (B, E), and FG vs. EG (C, F), with y-axes indicating pathways and x-axes showing relative proportions.

Table S1 Results of COG analyses predicted using PICRUSt

COG

	Freq1(EG)	Freq2(FG)	pValue	qValue	Difference between means	95% lower CI	95% upper CI
Poorly Characterized	10.31293082	7.983202183	9.55E-08	3.82E-07	2.329728641	2.006760022	2.652697261
Information Storage and Processing	18.93085705	23.87758078	0.000456	0.000912	-4.946723737	-6.530055863	-3.363391612
Metabolism	43.52931956	42.10042179	0.213538	0.284717	1.428897767	-1.364470512	4.222266046
Cellular Processes and Signaling	27.22689257	26.03879524	0.484912	0.484912	1.188097329	-3.313026257	5.689220915

	Freq1(EG)	Freq2(OG)	pValue	qValue	Difference between means	95% lower CI	95% upper CI
Poorly Characterized	10.31293082	10.61471381	0.209917	0.839669	-0.301782987	-0.811140705	0.20757473
Metabolism	43.52931956	44.54663992	0.350283	0.700566	-1.017320368	-3.776243341	1.741602606
Information Storage and Processing	18.93085705	18.3884946	0.448518	0.598023	0.542362447	-1.069583462	2.154308356
Cellular Processes and Signaling	27.22689257	26.45015167	0.636853	0.636853	0.776740908	-3.801215158	5.354696974

	Freq1(FG)	Freq2(OG)	pValue	qValue	Difference between means	95% lower CI	95% upper CI
Information Storage and Processing	23.87758078	18.3884946	1.95E-08	7.82E-08	5.489086184	4.402746351	6.575426017
Poorly Characterized	7.983202183	10.61471381	1.15E-07	2.30E-07	-2.631511629	-3.192627548	-2.070395709
Metabolism	42.10042179	44.54663992	5.09E-06	6.78E-06	-2.446218135	-3.20831145	-1.684124819
Cellular Processes and Signaling	26.03879524	26.45015167	0.412565	0.412565	-0.411356421	-1.454691074	0.631978232

Table S2 Results of KEGG analyses predicted using PICRUSt

KEGG							
	Freq1(EG)	Freq2(FG)	pValue	qValue	Difference between means	95% lower CI	95% upper CI
Cellular Processes	2.9731713	3.863933745	0.008271	0.049625	-0.890762445	-1.501000777	-0.280524113
Human Diseases	0.104765983	0.208863649	0.017566	0.052699	-0.104097666	-0.181270171	-0.026925161
Metabolism	80.16761332	78.97712756	0.045299	0.090599	1.19048576	0.030412523	2.350558997
Environmental Information Processing	2.241859171	2.706562599	0.107996	0.161994	-0.464703428	-1.05129456	0.121887703
Organismal Systems	0.378443551	0.440374756	0.113893	0.136672	-0.061931204	-0.142568196	0.018705787
Genetic Information Processing	14.13414667	13.80313769	0.45135	0.45135	0.331008983	-0.726204297	1.388222263

	Freq1(EG)	Freq2(OG)	pValue	qValue	Difference between means	95% lower CI	95% upper CI
Cellular Processes	2.9731713	6.282243099	2.01E-06	1.21E-05	-3.309071799	-4.083722986	-2.534420612
Environmental Information Processing	2.241859171	3.171281369	8.53E-05	0.000256	-0.929422198	-1.249803031	-0.609041366
Metabolism	80.16761332	78.27557408	0.000108	0.000217	1.892039246	1.182826262	2.60125223
Genetic Information Processing	14.13414667	11.64541477	0.00104	0.00156	2.488731898	1.411916505	3.565547292

Human Diseases	0.104765983	0.26071543	0.003272	0.003927	-0.155949447	-0.233122457	-0.078776438
Organismal Systems	0.378443551	0.364771251	0.716291	0.716291	0.0136723	-0.069368717	0.096713318

	Freq1(FG)	Freq2(OG)	pValue	qValue	Difference between means	95% lower CI	95% upper CI
Genetic Information Processing	13.80313769	11.64541477	1.33E-05	7.99E-05	2.157722915	1.448355396	2.867090434
Cellular Processes	3.863933745	6.282243099	2.33E-05	6.98E-05	-2.418309354	-3.270595139	-1.56602357
Human Diseases	0.208863649	0.26071543	0.029846	0.059691	-0.051851781	-0.097956767	-0.005746796
Organismal Systems	0.440374756	0.364771251	0.042193	0.06329	0.075603505	0.003017404	0.148189605
Environmental Information Processing	2.706562599	3.171281369	0.110226	0.132271	-0.46471877	-1.053692752	0.124255212
Metabolism	78.97712756	78.27557408	0.231593	0.231593	0.701553486	-0.508884851	1.911991823
